# Factors associated with myopia in the Portuguese child population: An epidemiological study

**DOI:** 10.1111/opo.13429

**Published:** 2024-11-28

**Authors:** Miguel Ángel Sánchez‐Tena, Clara Martinez‐Perez, Cristina Andreu‐Vázquez, Ana Roque, Cristina Alvarez‐Peregrina

**Affiliations:** ^1^ Department of Optometry and Vision, Faculty of Optics and Optometry Universidad Complutense de Madrid Madrid Spain; ^2^ ISEC LISBOA—Instituto Superior de Educação e Ciências Lisbon Portugal; ^3^ Faculty of Biomedical and Health Sciences Universidad Europea de Madrid Madrid Spain

**Keywords:** children, European, myopia, Portugal

## Abstract

**Purpose:**

Myopia, a leading cause of correctable visual impairment, is projected to affect nearly 50% of the global population by 2050, posing a significant public health challenge. Understanding its prevalence and associated factors, particularly in children, is crucial for devising prevention and intervention strategies. This study aims to determine the proportion of myopia in school‐aged children in Portugal and to examine the correlation between myopia occurrence and various environmental and genetic factors.

**Methods:**

A cross‐sectional epidemiological study was conducted on children aged from 5 to 17 years from nine schools in Lisbon, Portugal, between September 2020 and May 2021. It included optometric assessments to evaluate refractive status and binocular vision, as well as questionnaires about their lifestyles and parental myopia.

**Results:**

Out of 1992 participants enrolled, 12.7% of the children were found to be myopic. The proportion of myopia increased with age and was higher in girls. A significant association was observed between myopia and parental history, with the likelihood being higher if one or both parents were myopic. Engaging in outdoor activities was associated with a lower likelihood of myopia.

**Conclusions:**

The study found that 12.7% of the children in the study sample, aged 5–17 years, were myopic, indicating a significant association with familial history and limited outdoor activities. These insights highlight the need for targeted myopia screening and prevention strategies in the paediatric population.

**Key points**

- The proportion of myopia cases among children 5–17 years of age was 12.7%, with higher rates observed in older children and among girls.
- Children with a parental history of myopia had a significantly higher likelihood of being myopic, with this likelihood tripling if both parents were myopic, emphasising the role of familial factors.
- Spending more than 2.7 h per day outdoors was associated with a significantly lower likelihood of myopia, highlighting outdoor activity as a crucial preventive measure.

## INTRODUCTION

The prevalence of myopia is increasing globally and is considered the leading cause of correctable visual impairment in developed countries. By 2050, almost five billion people (nearly 50% of the world's population) may have myopia if current trends continue.^1^ The estimated number of people suffering from high myopia (6.0 dioptres or more) is a major concern. It is expected that by 2050, almost 10% of the global population (equivalent to approximately one billion individuals) will be diagnosed with this condition, leading to an increased risk of permanent visual impairment. This represents a 7.5‐fold increase from the statistics recorded in 2000.^1^

Additionally, the prevalence of myopia varies significantly across different regions. A systematic review published in 2016 reported that 1.6% of 5‐year‐old European children and 11.3% of Native North American children had myopia.^2^ The prevalence can be particularly high in some regions, such as East Asian countries, where up to 80% of the population may be affected.^3^

In Portugal, data on the prevalence and progression of myopia are very limited, which complicates ophthalmological care services.^4^ In a systematic review conducted in 2023, the authors found that the prevalence of refractive errors in Portugal was 31.9% between 2 and 59 years of age, meaning that at least between 2 and 4.5 million Portuguese have refractive errors. However, there is high heterogeneity among studies.^5^

The increase in the prevalence of myopia is driven by exposure to environmental factors present in urban and developed societies. Among the main risk factors are the time spent on near‐vision activities, less time spent outdoors, higher educational levels and family history.^6^ Among these factors, near work is most frequently associated with the development of myopia during school age. Near work encompasses tasks that require significant focus at close distances, such as reading, writing and computer and digital device use. Studies have shown that children with myopia tend to spend more time on activities like studying and reading and less time participating in outdoor sports, compared with children without myopia.^6^ Additionally, the problem of near work has been exacerbated by the increased use of digital devices at school age.^7^

Conversely, various studies have suggested positive effects of spending more time outdoors and making other behavioural changes on the onset and progression of myopia in children.^8^, ^9^, ^10^, ^11^, ^12^ Spending more time outdoors may prevent or delay the onset of myopia and reduce the effect of near work, which could potentially slow the rate of myopia progression.^12^ Although parental myopia is generally considered a risk factor for myopia in children, recent studies suggest that it may not necessarily be a genetic factor^6^ and that both genetic and environmental risk factors have an equally significant impact on myopia.^6^, ^13^, ^14^

The main objective of this study was to determine the proportion of myopia among school‐aged children within the Portuguese population. Additionally, the study seeks to examine the factors linked to myopia and explore potential correlations between its occurrence and these associated factors.

## METHODOLOGY

This was a cross‐sectional epidemiological analysis that gathered data from nine educational institutions in Lisbon, Portugal between September 2022 and May 2023. Schools were selected based on convenience sampling and participation was limited to students whose parents provided informed consent. The data collection was undertaken solely for research purposes, distinct from any routine screening activities. The study followed ethical standards in line with the Declaration of Helsinki and was approved by the ethics committee of Instituto Superior de Educação e Ciências (ISEC Lisbon) on 5 November 2021, with the identifier CE/2022/03/01. To be eligible for inclusion, participants had to be between 5 and 17 years old and their parents had to sign and understand the informed consent form.

### Clinical methods

The optometric assessment for each child consisted of a detailed questionnaire and a series of visual tests aimed at evaluating refractive status and binocular vision capabilities:

- Questionnaire: This tool recorded information about demographics (home city, age, gender and nationality), family eye history and lifestyles (including extracurricular activities, weekly hours spent on such activities, screen time, outdoor time and genetic factors).
- Optometric Examination: The established protocol started with the medical history and was followed by the following visual tests: assessment of both corrected and uncorrected visual acuity, objective refraction through non‐cycloplegic retinoscopy, subjective refraction and several binocular vision and accommodation tests (cover test, ocular movement analysis, Worth four dot test, convergence assessment, accommodative lag measurement, stereoscopic vision evaluation and colour perception).

To quantify outdoor time, an ordinal scale was developed based on the Clinical Myopia Profile^15^: that is, low (0–1.6 h daily), moderate (1.6–2.7 h daily) and high (>2.7 h daily). A similar scale was used for categorising time spent on near‐vision activities: low (0–2 h daily), moderate (2–3 h daily) and high (>3 h daily). Furthermore, screen time within these periods on near‐vision activities was classified into three ordinal segments <25%, between 25% and 50% and >50%. Additionally, to establish refractive error in children, the spherical equivalent (SE) was calculated using the formula: SE = sphere plus half of the cylinder value. Refractive errors were categorised as follows: hyperopia when SE was >+0.5 dioptres (D); myopia for SE < −0.5 D and emmetropia when SE ranged between −0.5 D and +0.5 D. Myopia was further divided into low (SE between −0.5 D and −3 D), moderate (SE between −3 D and −6 D) and high (SE ≤ −6 D), based on the American Academy of Optometry's standards.^16^

### Statistical analysis

Continuous variables are presented as mean and standard deviation (SD) or as median and interquartile range [Q1, Q3], depending on the distribution of the data (Shapiro–Wilk test). Categorical variables were described using absolute frequencies (*n*) and relative frequencies expressed as percentages. The proportion of myopia and its 95% confidence interval (CI) were calculated for the entire cohort of children and stratified by gender and age.

Differences in demographic characteristics, family history and lifestyle habits between children with and without myopia, as well as among different degrees of myopia, were evaluated using unpaired Student *t*‐tests/Mann–Whitney *U* tests (quantitative variables, according to their normality) or Chi‐squared tests (categorical variables).

Potential associations for myopia were investigated through logistic regression models. Univariate logistic regression models were constructed to evaluate the strength and direction of association between each factor and the presence or absence of myopia. Later, a multivariate logistic regression model was developed, considering significant factors identified in the univariate analysis and correcting for their potential interactions or confounding effects, to determine the adjusted association of each factor with myopia, while accounting for the influence of other significant factors.

Differences at the level of *p* < 0.05 were considered statistically significant. Statistical analyses were performed using the STATA v.17 software package (StataCorp LLC, stata.com).

## RESULTS

A total of 1992 children were enrolled in the study (48.8% boys), with an average age of 9.2 ± 2.6 years, of whom 25.7% were already wearing glasses or contact lenses to correct refractive errors before participating in the study.

### Proportion of myopia cases

The SE of the right eye was recorded in 1965 children (98.6% of the total children) who were classified based on refractive error as emmetropic (*n* = 647, 32.9%), myopic (*n* = 250, 12.7%) or hyperopic (*n* = 1068, 54.4%). Table 1 presents the SE and the percentage of myopia in the group according to age and sex.

**TABLE 1:** Spherical equivalent and percentage of myopia in the group of children, studied by age and sex.

	Boys	Girls	Overall
By age group			
5–8 years			
SE, RE (Median [Q1, Q3]; D)	+0.50 [0.00, +1.00]	+0.50 [0.00, +1.00]	+0.50 [0.00, +1.00]
*n* myopic/total *n*	25/417	32/456	57/873
Percentage of myopia	6.0%	7.0%	6.5%
(95% CI)	(4.1%, 8.7%)	(5.0%, 9.7%)	(5.1%, 8.4%)
9–12 years			
SE, RE (Median [Q1, Q3]; D)	+0.50 [0.00, + 0.75]	+0.50 [0.00, +0.75]	+0.50 [0.00, +0.75]
*n* myopic/total *n*	51/393	62/385	113/778
Percentage of myopia	13.0%	16.1%	14.5%
(95% CI)	(10.0%, 16.7%)	(12.8%, 20.1%)	(12.2%, 17.2%)
13–17 years			
SE, RE (Median [Q1, Q3]; D)	+0.25 [−0.50, +0.50]	0.00 [−0.88, +0.50]	+0.13 [−0.63, +0.50]
*n* myopic/total *n*	28/107	44/127	72/234
Percentage of myopia	26.2%	34.6%	30.8%
(95% CI)	(18.8%, 35.2%)	(26.9%, 43.3%)	(25.2%, 36.9%)
Overall^a^			
SE, RE (Median [Q1, Q3]; D)	+0.50 [0.00, +0.75]	+0.50 [0.00, +0.88]	+0.50 [0.00, +0.75]
*n* myopic/total *n*	107/958	143/1007	250/1965
Percentage of myopia	11.2%	14.2%	12.7%
(95% CI)	(9.3%. 913.3%)	(12.2%, 16.5%)	(11.3%, 14.3%)

Abbreviations: 95% CI, 95% confidence interval; D, dioptres; Q1, 1st quartile, 25th percentile; Q3, 3rd quartile, 75th percentile; RE, Right eye; SE, Spherical Equivalent.

^a^ Age data were missing for 80 children (41 boys and 39 girls) and so they are not represented in the age groups but are included in the overall totals.

### Factors associated with myopia

Table 2 compares the sociodemographic characteristics, family history and lifestyle habits of children with and without myopia. The median age of children with myopia was significantly higher than those without myopia and the proportion of girls was greater. A higher proportion of myopic children had one or both parents with myopia. Differences were also found between children with and without myopia with regard to the time spent on outdoor activities, near‐vision activities and the use of electronic devices.

**TABLE 2:** Comparison of characteristics, family history and lifestyle habits between children with and without myopia.

	Children with myopia (*n* = 250)	Children without myopia (*n* = 1715)	*p*‐Value^a^
Age (years, median [Q1, Q3])	10 [9, 13]	9 [7, 10]	<0.001
Gender			
Male (*n*, %)	107 (42.8%)	851 (49.6%)	0.04
Female (*n*, %)	143 (57.2%)	864 (50.4%)	
Parents with myopia			
None	66 (28.5%)	803 (50.4%)	<0.001
One (mother or father)	112 (48.3%)	620 (38.9%)	
Both	54 (23.3%)	170 (10.7%)	
Time spent outdoors			
Low (0–1.6 h)	73 (30.2%)	406 (24.7%)	0.048
Moderate (1.6–2.7 h)	149 (61.6%)	1031 (62.6%)	
High (>2.7 h)	20 (8.3%)	210 (12.7%)	
Time spent on near‐vision activities			
Low (0–2 h)	78 (32.5%)	685 (41.7%)	<0.001
Moderate (2–3 h)	113 (47.1%)	760 (46.3%)	
High (>3 h)	49 (20.4%)	197 (12.0%)	
Digital device usage			
<25% time spent in near‐vision activities	59 (24.4%)	574 (34.9%)	<0.001
25%–50% time spent in near‐vision activities	109 (45.0%)	782 (47.5%)	
>50% time spent in near‐vision activities	74 (30.6%)	289 (17.6%)	

*Note*: Number of children with missing data for each variable: age: 80; parental myopia: 140; outdoor time: 76; time spent on near‐vision activities: 83; digital device usage: 78.

Abbreviations: 95% CI, 95% confidence interval; D, dioptres; Q1, 1st quartile, 25th percentile; Q3, 3rd quartile, 75th percentile.

^a^ Comparison between children with and without myopia using Mann–Whitney U test (Age, non‐normally distributed quantitative variable) or Chi‐squared tests (categorical variables).

**FIGURE 1:**
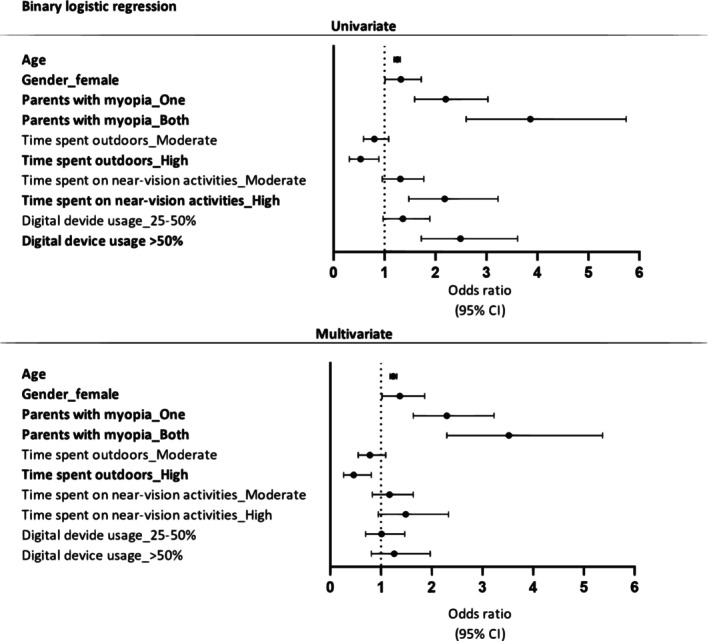
Results of univariate and multivariate binary logistic regression to determine the factors associated with myopia and their strength of association. Univariate logistic regressions used children with known data for each variable. The multivariate model included 1760 children with complete data for all variables.

Figure 1 shows the results of the binary logistic regression models for factors associated with the likelihood of myopia. The univariate model shows the strength and direction of the association (odds ratio and its 95% CI) for each of the factors studied individually, while the multivariate model adjusts the effect of each factor by the other associated factors. The likelihood of myopia increased by 24% (OR: 1.24; 95% CI: 1.18–1.31) for each additional year of age and was 37% higher (OR: 1.37; 95% CI: 1.02–1.86) in girls than boys. If the mother or father was myopic (OR: 2.30; 95% CI: 1.64–3.23), then the likelihood of the child having myopia doubled, and this likelihood was more than tripled when both parents were myopic (OR: 3.52; 95% CI: 2.30–5.37) compared with children whose parents were not myopic. Time spent on outdoor activities was a protective factor against myopia. The likelihood of myopia halved in children who spent more than 2.7 h a day doing outdoor activities (OR: 0.46; 95% CI: 0.26–0.81). However, although the univariate model showed a significant association between the time dedicated to near‐vision activities and the use of electronic devices, the multivariate model reveals that these factors were not significantly associated with myopia.

### Degree of myopia

Out of the 250 children with myopia, 86.8% (*n* = 217) had low levels of myopia, while moderate and high myopia were present in 11.7% (*n* = 29) and 1.6% (*n* = 4) of the children, respectively. Table 3 compares the characteristics of children with different degrees of myopia. Children with moderate myopia tended to be older than those with low myopia. The proportion of children with two myopic parents was significantly higher in individuals with moderate myopia than in those with low myopia. No significant differences were found with sex or in the time spent undertaking outdoor activities, near‐vision activities or using electronic devices.

**TABLE 3:** Comparison of characteristics, family history and lifestyle habits among children with different degrees of myopia.

	Low myopia (*n* = 217)	Moderate myopia (*n* = 29)	High myopia (*n* = 4)	*p*‐Value^a^
Age (years, median [Q1, Q3])	10 [8, 13]	11 [9, 15]	14.5 [10, 15.5]	0.08
Gender				
Male (*n*, %)	89 (41.0%)	16 (55.2%)	2 (50.0%)	0.15
Female (*n*, %)	128 (59.0%)	13 (44.8%)	2 (50.0%)	
Parents with myopia				
None	62 (31.0%)	4 (13.8%)	0	0.008
One (mother or father)	98 (49.0%)	12 (41.4%)	2 (50.0%)	
Both	40 (20.0%)	13 (44.8%)	2 (50.0%)	
Time spent outdoors				
Low (0–1.6 h)	60 (28.7%)	13 (44.8%)	0	0.21
Moderate (1.6–2.7 h)	132 (63.1%)	14 (48.3%)	3 (75.0%)	
High (>2.7 h)	17 (8.1%)	2 (6.9%)	1 (25.0%)	
Time spent on near‐vision activities				
Low (0–2 h)	70 (33.7%)	7 (25.0%)	1 (25.0%)	0.62
Moderate (2–3 h)	97 (46.6%)	14 (50.0%)	2 (50.0%)	
High (>3 h)	41 (19.7%)	7 (25.0%)	1 (25.0%)	
Digital device usage				
<25% time spent in near‐vision activities	54 (25.8%)	4 (13.8%)	1 (25.0%)	0.25
25%–50% time spent in near‐vision activities	94 (45.0%)	13 (44.8%)	2 (50.0%)	
>50% time spent in near‐vision activities	61 (29.2%)	12 (41.4%)	1 (25.0%)	

*Note:* Number of children with missing data for each variable: age: 80; parental myopia: 140; outdoor time: 76; time spent on near‐vision activities: 83; digital device usage: 78.

Abbreviations: Q1: 1st quartile, 25th percentile; Q3: 3rd quartile, 75th percentile.

^a^ Comparison between children with low and moderate myopia using Mann–Whitney *U* test (age, non‐normally distributed quantitative variable) or Chi‐Squared tests (categorical variables). High myopia could not be compared due to the low number of children (*n* = 4).

## DISCUSSION

This article is the first to analyse the proportion of myopia in almost 2000 school‐age children in Portugal and its relationship with associated factors. Thus, it was found that 12.7% of participants (ages 5–17) were myopic.

In the context of Portugal, previous research has concentrated on studying the prevalence of refractive errors, including hyperopia, within the population. As a result, the data obtained from these studies cannot be compared with the present results. In 2023, Alves Carneiro and González‐Méijome conducted a systematic review and meta‐analysis to examine the epidemiological data on the prevalence of refractive error in Portugal. The study reviewed nine other studies conducted between 1999 and 2017 and the findings indicated that the prevalence of refractive error in Portugal was nearly 32%. However, the authors suggested that more consistent sources and studies were necessary to obtain more precise estimates since the analysed studies showed significant variation and random effects. The use of a random effects model accounts for variability both within and between studies, acknowledging that differences in study designs, populations and settings may lead to variations in the effect sizes observed across studies.^5^ In the same year, a further study on refractive errors in Portugal indicated that 69.3% of the Portuguese population between 6 and 29 years of age had myopia. However, this study's small sample size, with only 348 optometry records analysed and the bias of examining only people who presented to optometrists, rather than a general and healthy population, makes these results incomparable with the previous study's findings. Therefore, these results do not represent the rates of myopia in Portugal with confidence.^17^

Regarding other countries, in 2019, the International Myopia Institute established a standardised set of terminology, definitions and myopia thresholds.^18^ When comparing the percentage of myopia in school‐aged children from the current study in Portugal with other countries, it was found to be lower than in China (23.3%),^19^ Denmark (17.9%),^20^ France (19.6%)^21^ and Spain (20.4%),^22^ but similar to the percentages found in India (13.1%),^23^ Colombia (11.2%)^24^ and Australia (14.8%).^25^ These differences in the percentage of myopia may be due to differences in lifestyle and educational levels. Thus, in those countries with more limited education, the prevalence of myopia is low (less than 10% of myopia in young adults). In contrast, in countries with Western‐style education, the prevalence is between 10 and 60%. In developed countries in East and Southeast Asia, where education is intense and outdoor time is low, the prevalence of myopia is high (70–90%), particularly in urban areas.^26^ The very high prevalence rates that are now typical of developed countries in East and Southeast Asia, compared with much more moderate prevalence rates observed in Western countries, cannot simply be attributed to more years of education but are probably related to the intensity of schooling, an earlier start to a large amount of near work and much less time being spent outdoors.

Although recent studies have found similar associations between reading and myopia, with specific research indicating that an increase of 1 h of reading per week is associated with a 5% higher likelihood of school‐age myopia,^27^ the present results do not reflect this trend. Rather, we agree with studies that have reported contradictory results,^28^, ^29^ suggesting that the discrepancy may stem from different methodologies for measuring near work. For example, Ip et al.^28^ analysed the intensity and distance of reading, rather than the duration, finding that prolonged reading sessions and proximity to the reading material increased the risk of myopia. On the other hand, Saw et al.^29^ identified a significant relationship with the number of books read, but not with the number of reading hours per week. This variety of approaches highlights the need for a standardised and objective method to quantify the variables of near work and longitudinal studies could provide clarity to these relationships.

Regarding the use of digital devices, the meta‐analysis by Foreman et al.^30^ indicated an association with myopia when combining cross‐sectional and prospective studies, but the heterogeneity of these studies warrants cautious interpretation. A previous meta‐analysis found that each additional hour of near‐vision work increased the risk of myopia by 2%.^31^ Given that smart devices are used for longer periods and at shorter distances than other near‐vision activities, it is hypothesised that they contribute to the development of myopia. However, the present findings do not show a clear association. This lack of association may be attributed to challenges in accurately quantifying the use of these devices and near‐vision activities, as well as the complexity of accounting for multiple potential confounding factors in cross‐sectional studies.

One of the most well‐documented risk factors is having parents with myopia. Although the consistent impact of parental myopia can be explained because myopic parents transmit genetic variants that predispose their children to myopia, it is also likely that myopic parents, on average, have a higher level of education.^6^ This implies that environmental risk factors may also be involved, as concluded by Enthoven et al.^14^

In line with the present findings, the existing literature suggests that spending time outdoors could have a protective effect against myopia.^7^ The mechanism of this association is not yet well understood, but two theories have been proposed: one is the ‘light‐dopamine theory’, which highlights that increased light intensity while spending time outdoors protects against myopia due to greater dopamine release.^32^, ^33^, ^34^ Alternatively, the ‘vitamin D theory’ hypothesises that increased ultraviolet light triggers the stimulation of vitamin D production, with a direct protective effect against the development of myopia.^35^, ^36^, ^37^, ^38^ A recent meta‐analysis by Tang et al. reported that a lower concentration of 25‐hydroxyvitamin D (25(OH)D) is associated with a higher risk of myopia.^39^

This study has some limitations. First, the data were obtained only from the Lisbon region, although it is an urbanised and densely populated city with different study areas. Second, refraction was determined using non‐cycloplegic objective refraction, which can lead to a misclassification of refractive error with an overestimation of myopia. This approach was chosen based on practical constraints. Despite this limitation, the large sample size of nearly 2000 children enhances the robustness and reliability of the findings, providing a valuable snapshot of the proportion of myopia cases and associated factors within this population.

Based on these findings, we recommend implementing comprehensive vision screening programmes for school‐aged children in Portugal. These programmes should initiate early screenings, ideally before children start primary school and continue with regular annual assessments to monitor changes in vision, particularly among high‐risk groups such as those with a family history of myopia. Additionally, educational initiatives should be developed to inform parents and educators about the significance of promoting outdoor activities and moderating near work, such as reading and digital device usage, as preventive measures against myopia. Moreover, it is essential to provide appropriate follow‐up care, including corrective measures and regular monitoring for children identified with significant refractive errors, to manage and mitigate the potential impact of visual impairments effectively.

In conclusion, the proportion of myopia among children 5–17 years of age in the study sample was 12.7%. The investigation identified significant associations between myopia and factors such as parental history of myopia and reduced time spent on outdoor activities. Additionally, while the use of digital devices and near‐vision activities were considered, no clear association with myopia was established in this cross‐sectional study. These findings highlight the importance of promoting outdoor activities and considering family history when determining prevention strategies for myopia. Future research should focus on longitudinal studies to explore further the causal relationships and potential interventions for myopia prevention. This study underscores the need for targeted screening and early interventions to mitigate the increasing burden of myopia in the paediatric population in Portugal.

## AUTHOR CONTRIBUTIONS

**Miguel Ángel Sánchez‐Tena:** Conceptualization (equal); methodology (equal); supervision (equal); validation (equal); writing – review and editing (equal). **Clara Martinez‐Perez:** Data curation (equal); investigation (equal); project administration (equal); writing – original draft (equal). **Cristina Andreu‐Vázquez:** Formal analysis (equal); software (equal); writing – original draft (equal). **Ana Roque:** Data curation (equal); writing – review and editing (equal). **Cristina Alvarez‐Peregrina:** Conceptualization (equal); investigation (equal); methodology (equal); supervision (equal); writing – review and editing (equal).

## FUNDING INFORMATION

This research received no specific grant from any funding agency in the public, commercial or not‐for‐profit sectors.

## CONFLICT OF INTEREST STATEMENT

The authors report no conflicts of interest and have no proprietary interest in any of the materials mentioned in this article.

## HUMAN SUBJECTS AND INFORMED CONSENT

This research was reviewed by an independent ethical review board and conforms with the principles and applicable guidelines for the protection of human subjects in biomedical research.

## References

[1] Holden BA , Fricke TR , Wilson DA , Jong M , Naidoo KS , Sankaridurg P , et al. Global prevalence of myopia and high myopia and temporal trends from 2000 through 2050. Ophthalmology. 2016;123:1036–1042. 26875007 10.1016/j.ophtha.2016.01.006

[2] Rudnicka AR , Kapetanakis VV , Wathern AK , Logan NS , Gilmartin B , Whincup PH , et al. Global variations and time trends in the prevalence of childhood myopia, a systematic review and quantitative meta‐analysis: implications for aetiology and early prevention. Br J Ophthalmol. 2016;100:882–890. 26802174 10.1136/bjophthalmol-2015-307724 PMC4941141

[3] Morgan IG , French AN , Ashby RS , Guo X , Ding X , He M , et al. The epidemics of myopia: aetiology and prevention. Prog Retin Eye Res. 2018;62:134–149. 28951126 10.1016/j.preteyeres.2017.09.004

[4] de Saude DG . Estrategia Nacional para a Saude da Visao. 2018 1126. Accessed September 23, 2023. https://revistas.rcaap.pt/oftalmologia/article/view/15414

[5] Alves Carneiro VL , González‐Méijome JM . Prevalence of refractive error in Portugal – a systematic review and meta‐analysis. J Optom. 2023;16:182–188. 36050229 10.1016/j.optom.2022.07.003 PMC10323180

[6] Morgan IG , Wu PC , Ostrin LA , Tideman JWL , Yam JC , Lan W , et al. IMI risk factors for myopia. Invest Ophthalmol Vis Sci. 2021;62:3. 10.1167/iovs.62.5.3 PMC8083079 33909035

[7] American Optometric Association (AOA) . The 21st century child: increased technology use may lead to future eye health and vision issues (online). St Louis: American Optometric Association; 2015.

[8] Xiong S , Sankaridurg P , Naduvilath T , Zang J , Zou H , Zhu J , et al. Time spent in outdoor activities in relation to myopia prevention and control: a meta‐analysis and systematic review. Acta Ophthalmol. 2017;95:551–566. 28251836 10.1111/aos.13403 PMC5599950

[9] Rose KA , Morgan IG , Ip J , Kifley A , Huynh S , Smith W , et al. Outdoor activity reduces the prevalence of myopia in children. Ophthalmology. 2008;115:1279–1285. 18294691 10.1016/j.ophtha.2007.12.019

[10] Read SA , Collins MJ , Vincent SJ . Light exposure and eye growth in childhood. Invest Ophthalmol Vis Sci. 2015;56:6779–6787. 26567790 10.1167/iovs.14-15978

[11] Guggenheim JA , Northstone K , McMahon G , Ness AR , Deere K , Mattocks C , et al. Time outdoors and physical activity as predictors of incident myopia in childhood: a prospective cohort study. Invest Ophthalmol Vis Sci. 2012;53:2856–2865. 22491403 10.1167/iovs.11-9091 PMC3367471

[12] He M , Xiang F , Zeng Y , Mai J , Chen Q , Zhang J , et al. Effect of time spent outdoors at school on the development of myopia among children in China: a randomized clinical trial. JAMA. 2015;314:1142–1148. 26372583 10.1001/jama.2015.10803

[13] Tedja MS , Haarman AEG , Meester‐Smoor MA , Kaprio J , Mackey DA , Guggenheim JA , et al. IMI – myopia genetics report. Invest Ophthalmol Vis Sci. 2019;60:M89–M105. 30817828 10.1167/iovs.18-25965 PMC6892384

[14] Enthoven CA , Tideman JWL , Polling JR , Tedja MS , Raat H , Iglesias AI , et al. Interaction between lifestyle and genetic susceptibility in myopia: the generation R study. Eur J Epidemiol. 2019;34:777–784. 30945054 10.1007/s10654-019-00512-7 PMC6602996

[15] Gifford K . Myopia profile: an information resource for optometrists. 2013 Accessed July 1, 2023: https://www.myopiaprofile.com/store/

[16] Goss DA , Grosvenor TP , Keller JT , Marsh‐Tootle W , Norton TT , Zadnik K . Care of the patient with myopia: optometric clinical practice guideline. St Louis, Missouri: American Optometric Association; 2006.

[17] Carneiro VLA , González‐Méijome JM . Prevalence of refractive error within a Portuguese sample of optometric records. J Optom. 2023;16:245–251. 37164811 10.1016/j.optom.2023.04.001 PMC10518762

[18] Flitcroft DI , He M , Jonas JB , Jong M , Naidoo K , Ohno‐Matsui K , et al. IMI – defining and classifying myopia: a proposed set of standards for clinical and epidemiologic studies. Invest Ophthalmol Vis Sci. 2019;60:M20–M30. 30817826 10.1167/iovs.18-25957 PMC6735818

[19] Lin Z , Gao TY , Vasudevan B , Jhanji V , Ciuffreda KJ , Zhang P , et al. Generational difference of refractive error and risk factors in the Handan offspring myopia study. Invest Ophthalmol Vis Sci. 2014;55:5711–5717. 25097244 10.1167/iovs.13-13693

[20] Lundberg K , Suhr Thykjaer A , Søgaard Hansen R , Vestergaard AH , Jacobsen N , Goldschmidt E , et al. Physical activity and myopia in Danish children‐the CHAMPS eye study. Acta Ophthalmol. 2018;96:134–141. 28671340 10.1111/aos.13513

[21] Matamoros E , Ingrand P , Pelen F , Bentaleb Y , Weber M , Korobelnik JF , et al. Prevalence of myopia in France: a cross‐sectional analysis. Medicine (Baltimore). 2015;94:e1976. 10.1097/MD.0000000000001976 26559276 PMC4912270

[22] Alvarez‐Peregrina C , Martinez‐Perez C , Villa‐Collar C , González‐Pérez M , González‐Abad A , Sánchez‐Tena MÁ , et al. The prevalence of myopia in children in Spain: an updated study in 2020. Int J Environ Res Public Health. 2021;18:12375. 10.3390/ijerph182312375 34886101 PMC8656604

[23] Saxena R , Vashist P , Tandon R , Pandey RM , Bhardawaj A , Menon V , et al. Prevalence of myopia and its risk factors in urban school children in Delhi: the North India Myopia study (NIM study). PLoS One. 2015;10:e0117349. 10.1371/journal.pone.0117349 25719391 PMC4342249

[24] Galvis V , Tello A , Otero J , Serrano AA , Gómez LM , Castellanos Y . Refractive errors in children and adolescents in Bucaramanga (Colombia). Arq Bras Oftalmol. 2017;80:359–363. 29267570 10.5935/0004-2749.20170088

[25] French AN , Morgan IG , Mitchell P , Rose KA . Risk factors for incident myopia in Australian schoolchildren: the Sydney adolescent vascular and eye study. Ophthalmology. 2013;120:2100–2108. 23672971 10.1016/j.ophtha.2013.02.035

[26] Ang M , Wong TY . Updates on myopia. Singapore: SpringerOpen; 2020.

[27] Paudel P , Ramson P , Naduvilath T , Wilson D , Phuong HT , Ho SM , et al. Prevalence of vision impairment and refractive error in school children in Ba Ria – Vung tau province, Vietnam. Clin Exp Ophthalmol. 2014;42:217–226. 24299145 10.1111/ceo.12273 PMC4291105

[28] Ip JM , Saw SM , Rose KA , Morgan IG , Kifley A , Wang JJ , et al. Role of near work in myopia: findings in a sample of Australian school children. Invest Ophthalmol Vis Sci. 2008;49:2903–2910. 18579757 10.1167/iovs.07-0804

[29] Saw SM , Chua WH , Hong CY , Wu HM , Chan WY , Chia KS , et al. Nearwork in early‐onset myopia. Invest Ophthalmol Vis Sci. 2002;43:332–339. 11818374

[30] Foreman J , Salim AT , Praveen A , Fonseka D , Ting DSW , Guang He M , et al. Association between digital smart device use and myopia: a systematic review and meta‐analysis. Lancet Digit Health. 2021;3:e806–e818. 34625399 10.1016/S2589-7500(21)00135-7

[31] Huang HM , Chang DS , Wu PC . The association between near work activities and myopia in children‐a systematic review and meta‐analysis. PLoS One. 2015;10(10):e0140419. 10.1371/journal.pone.0140419 26485393 PMC4618477

[32] Megaw PL , Boelen MG , Morgan IG , Boelen MK . Diurnal patterns of dopamine release in chicken retina. Neurochem Int. 2006;48:17–23. 16188347 10.1016/j.neuint.2005.08.004

[33] McCarthy D , Lueras P , Bhide PG . Elevated dopamine levels during gestation produce region‐specific decreases in neurogenesis and subtle deficits in neuronal numbers. Brain Res. 2007;1182:11–25. 17950709 10.1016/j.brainres.2007.08.088 PMC2141544

[34] Li W , Lan W , Yang S , Liao Y , Xu Q , Lin L , et al. The effect of spectral property and intensity of light on natural refractive development and compensation to negative lenses in Guinea pigs. Invest Ophthalmol Vis Sci. 2014;55:6324–6332. 25277235 10.1167/iovs.13-13802

[35] Choi JA , Han K , Park YM , La TY . Low serum 25‐hydroxyvitamin D is associated with myopia in Korean adolescents. Invest Ophthalmol Vis Sci. 2014;55:2041–2047. 24699557 10.1167/IOVS.13-12853

[36] Guggenheim JA , Williams C , Northstone K , Howe LD , Tilling K , St Pourcain B , et al. Does vitamin D mediate the protective effects of time outdoors on myopia? Findings from a prospective birth cohort. Invest Ophthalmol Vis Sci. 2014;55:8550–8558. 25406278 10.1167/iovs.14-15839 PMC4280087

[37] Mutti DO , Marks AR . Blood levels of vitamin D in teens and young adults with myopia. Optom Vis Sci. 2011;88:377–382. 21258262 10.1097/OPX.0b013e31820b0385 PMC3044787

[38] Tideman JW , Polling JR , Voortman T , Jaddoe VWV , Uitterlinden AG , Hofman A , et al. Low serum vitamin D is associated with axial length and risk of myopia in young children. Eur J Epidemiol. 2016;31:491–499. 26955828 10.1007/s10654-016-0128-8 PMC4901111

[39] Tang SM , Lau T , Rong SS , Yazar S , Chen LJ , Mackey DA , et al. Vitamin D and its pathway genes in myopia: systematic review and meta‐analysis. Br J Ophthalmol. 2019;103:8–17. 30018147 10.1136/bjophthalmol-2018-312159

